# Exploring type I interferon pathway: virulent vs. attenuated strain of African swine fever virus revealing a novel function carried by MGF505-4R

**DOI:** 10.3389/fimmu.2024.1358219

**Published:** 2024-03-11

**Authors:** Juliette Dupré, Mireille Le Dimna, Evelyne Hutet, Pascal Dujardin, Aurore Fablet, Aurélien Leroy, Isabelle Fleurot, Grégory Karadjian, Ferdinand Roesch, Ignacio Caballero, Olivier Bourry, Damien Vitour, Marie-Frédérique Le Potier, Grégory Caignard

**Affiliations:** ^1^Unité Mixte de Recherche (UMR) VIROLOGIE, Institut National Recherche pour l’Agriculture, l’Alimentation et l’Environnement (INRAE), École Nationale Vétérinaire d’Alfort (ENVA), Agence Nationale de Sécurité Sanitaire de l'Alimentation, de l'Environnement et du Travail (ANSES) Laboratoire de Santé Animale, Université Paris-Est, Maisons-Alfort, France; ^2^Unité Virologie Immunologie Porcines, Laboratoire de Ploufragan-Plouzané-Niort, ANSES, Ploufragan, France; ^3^UMR 1282 Infectiologie et santé publique (ISP), INRAE Centre Val de Loire, Nouzilly, France; ^4^UMR Biologie moléculaire et Immunologie Parasitaires (BIPAR), ENVA-INRAE-ANSES, Laboratoire de Santé Animale, Maisons-Alfort, France

**Keywords:** African Swine Fever Virus, IFN, viral immune evasion, virulence factor, TRAF3

## Abstract

African swine fever virus represents a significant reemerging threat to livestock populations, as its incidence and geographic distribution have surged over the past decade in Europe, Asia, and Caribbean, resulting in substantial socio-economic burdens and adverse effects on animal health and welfare. In a previous report, we described the protective properties of our newly thermo-attenuated strain (ASFV-989) in pigs against an experimental infection of its parental Georgia 2007/1 virulent strain. In this new study, our objective was to characterize the molecular mechanisms underlying the attenuation of ASFV-989. We first compared the activation of type I interferon pathway in response to ASFV-989 and Georgia 2007/1 infections, employing both *in vivo* and *in vitro* models. Expression of *IFN-α* was significantly increased in porcine alveolar macrophages infected with ASFV-989 while pigs infected with Georgia 2007/1 showed higher IFN-α than those infected by ASFV-989. We also used a medium-throughput transcriptomic approach to study the expression of viral genes by both strains, and identified several patterns of gene expression. Subsequently, we investigated whether proteins encoded by the eight genes deleted in ASFV-989 contribute to the modulation of the type I interferon signaling pathway. Using different strategies, we showed that MGF505-4R interfered with the induction of IFN-α/β pathway, likely through interaction with TRAF3. Altogether, our data reveal key differences between ASFV-989 and Georgia 2007/1 in their ability to control IFN-α/β signaling and provide molecular mechanisms underlying the role of MGF505-4R as a virulence factor.

## Introduction

African Swine Fever (ASF) represents a significant health risk due to its ability to cause severe and often fatal disease in domestic pigs and wild boars, with no efficient vaccine or treatment. Due to the ensuing high socio-economic burden and impact on animal health, ASF is a listed disease by the World Organisation for Animal Health (WOAH). Therefore, ASF remains a significant challenge for the worldwide pig farming sector, casting a pervasive shadow over porcine populations. The causative agent, African Swine Fever Virus (ASFV) is a large enveloped virus belonging to the *Asfarviridae* family. Transmission can occur through direct contacts between animals (1, 2), by aerosol (3) but also via soft ticks of the *Ornithodoros* genus (4–6). Due to its high resistance in the environment, ASFV can also be indirectly transmitted to pig or wild boar through the ingestion of infected pork or exposure to contaminated tools or clothes. Virulent strains induce hemorrhagic fever with mortality rates up to nearly 100% in domestic pigs and wild boars, while adult African wild suids appear asymptomatic (7–11). In 2007, the ASFV Georgia 2007/1 strain has emerged in Georgia (12) and has spread all over the European continent, entering the European Union in 2014, then China and South-Eastern Asia in 2018, and the Caribbean in 2021. This major panzootic of ASFV has unfolded, impacting almost every continent and leading to devastating economic and social consequences (13). ASFV harbors a double-stranded DNA (dsDNA) genome of unparalleled complexity, with genes that encode between 150 and 170 proteins, depending on the isolate. This variation is mainly due to the loss or gain of genes within the Multigene families (MGF) (14). Previous works suggest that genes within MGF360 and MGF505 are crucial in the modulation of the type I Interferon (IFN-α/β) signaling pathway (15–17). Moreover, attenuated ASFV strains, exhibiting the loss or truncation of various MGF360 and 505 genes, are more sensitive to IFN-α in comparison to their virulent counterparts (18–21).

Among the arsenal of host defense mechanisms, IFN-α/β emerge as sentinel molecules in the early antiviral response. Indeed, IFN-α/β orchestrate a multifaceted defense strategy by inducing an array of antiviral proteins, encoded by ISGs (Interferon stimulated genes), that inhibit viral replication and spread (22). The critical role of IFN-α/β for the establishment of an efficient antiviral response against ASFV has also been documented (23, 24). Their synthesis are based on the recognition of specific molecular motifs known as PAMPs (pathogen-associated molecular patterns) by either membrane receptors or cytoplasmic sensors, collectively termed PRRs (pattern recognition receptors). The PRRs involved in the detection of ASFV are only partially characterized to date, with cGAS (Cyclic GMP-AMP synthase) emerging as the most extensively studied among them (25–28). cGAS is a sensor capable of detecting dsDNA of over 40 base pairs. Upon binding to dsDNA, cGAS undergoes a conformational change, subsequently catalyzing the production of cGAMP (2’,3’-cyclic GMP-AMP). This cGAMP molecule is then recognized by the transmembrane receptor STING (stimulator of interferon genes), which is embedded in the endoplasmic reticulum (ER) membrane. Binding of cGAMP to STING triggers its conformational change, resulting in oligomerization and translocation of STING from the ER to the Golgi apparatus. STING subsequently recruits TBK1 (TANK-binding kinase 1), which in turn activates both STING and IRF3 (Interferon regulatory factor 3). This activation leads to IRF3 dimerization and its nuclear translocation, enabling the transcription of *IFN-α/β* genes (29).

The significant enrichment of AT sequences in the genome of ASFV allows these regions to be also recognized by RNA Polymerase III and the transcripts produced are subsequently detectable by RIG-I (30). RIG-I (retinoic acid inducible gene I) is a cytosolic sensor that recognizes the free 5’-triphosphate ends of exogenous RNAs, which differ from cellular RNAs, usually capped. Uppon activation, RIG-I is taken over by MAVS (mitochondrial antiviral signaling protein) via its CARD domains. This leads to dimerization of MAVS, which then associates with TRAF3 (tumor necrosis factor (TNF) receptor-associated factor 3) and TANK (TRAF family member-associated NF-kappa B activator). The latter can recruit the IKK (Inhibitory protein κB kinase) and TBK1 kinases, inducing activation of the IRF3 and IRF7 (and NF-kB via IKK) transcription factors and, ultimately, the transcription of *IFN-α/β* genes. Altogether, the RIG-I-TBK1 axis also appears to be activated in response to ASFV infection (31).

As known for other viruses, the pathogenesis of ASFV involves a complex interplay between viral factors and the host’s intricate immune defenses, making it imperative to dissect the molecular intricacies that govern this dynamic interaction. In our previous report (32), we described and characterized the protective properties of the newly thermo-attenuated strain ASFV-989 in pigs against an experimental infection of its parental virulent Georgia 2007/1 strain. The ASFV-989 strain is distinguished by the deletion of eight genes within the MGF360 and MGF505 regions (MGF360-12L, -13L, -14L, MGF505-1R, -2R, -3R, -4R and G_ACD_00520). The molecular mechanisms underlying the attenuation of ASFV-989 remain to be established. In this study, we first compared the induction of IFN-α/β pathway in response to ASFV-989 and Georgia 2007/1 infections, employing both *in vivo* and *in vitro* models. We used a medium-throughput transcriptomic approach to examine the expression of viral genes in both strains, uncovering various patterns of gene expression. Subsequently, we explored whether proteins encoded by the eight genes deleted in ASFV-989 play a role in the modulation of the IFN-α/β signaling pathway. Using different strategies, we provide unprecedented evidence for the crucial role of MGF505-4R in counteracting the induction of the IFN-α/β response and suggest that this inhibition involves a key actor of the IFN signaling, TRAF3. Interestingly, the conserved fragment (MGF505-4R^109-506^) in ASFV-989 was shown to impair MGF505-4R inhibitory function, potentially contributing to the attenuation of this new potential vaccine candidate.

## Materials and methods

### Viruses and cells

The virulent Georgia 2007/1 strain was isolated from domestic pigs in Georgia in 2007 (12). The attenuated ASFV-989 strain was obtained after thermo-attenuation of the Georgia 2007/1 as previously described (32). Both strains were grown in primary porcine alveolar macrophages (PAMs) cultures and used after three passages for the Georgia 2007/1 strain and one passage for the ASFV-989 strain for pig inoculation. For the *in vivo* study, viruses were diluted in RPMI medium to adjust the inoculation dose to 1 x 10^3^ hemadsorbing dose 50% (HAD50) per pig for intramuscular (i.m.) inoculation. Inoculation doses for animal experiments were confirmed by back titration. PAMs were maintained in RPMI 1640 medium with 10% fetal bovine serum, 1% L-glutamine, 1% penicillin/streptomycin at 37°C and 5% CO_2_. HEK-293T and HeLa cells were maintained in DMEM medium containing 10% fetal bovine serum, 1% sodium pyruvate and 1% penicillin/streptomycin at 37°C and 5% CO_2_.

### Pig experiments

The blood samples were collected in a previous *in vivo* study (32) that had been approved by the ethics committee N°16 (authorization number 19-018#19585) and authorized by the French Ministry of Research (project n°2019030418445731). The SPF (specific pathogen free) pigs were inoculated using intramuscular (i.m.) route for both strains, with 6 pigs inoculated with Georgia 2007/1 and 17 pigs inoculated with ASFV-989. EDTA-blood and serum samples were collected at day 3 and 5 post inoculation for virological and IFN assays, respectively. Assessment of the ASFV viremia in infected pigs was performed with a pan-ASFV real-time PCR, as previously described (33), using DNA extracted from 100 μL of EDTA blood samples with the NucleoSpin^®^ 8 Virus (Macherey-Nagel, Düren, Germany). The ASFV genomic load was determined by means of a standard viral range (with a known HAD_50_ titer) diluted in negative blood. The results were expressed as equivalent (eq) HAD_50_/mL of blood. The protein level of IFN-α was determined by a sandwich ELISA as previously described (34). Microtiter plates (Nunc, Roskilde, Denmark) were coated overnight at room temperature (RT) with K9 IFN-α mAb at 7.5 μg/mL in PBS, then blocked for 1 hour (h) at 37°C in PBS containing 5% BSA and 0.05% Tween 20. After three washes in PBS containing 0.05% Tween 20, plates containing samples and recombinant IFN-α protein (R&D Systems) standard range were incubated for 2h at RT. After five washes, biotinylated-F17 IFN-α mAb (0.5µg/ml) was added for 2h at RT. Following three washes, peroxidase-conjugated ExtrAvidin (Sigma–Aldrich) was then added for 1h at 37°C. Peroxidase activity was measured using the tetramethilbenzidine substrate (Sigma–Aldrich) at 450 nm.

### Infection experiments in PAMs

PAMs were plated in 24-well plates (1.2 x 10^6^ cells per well). After 4h, cells were infected with either the Georgia 2007/1 or ASFV-989 strains with a multiplicity of infection (MOI) of 2. The inoculum was removed after one hour of incubation, and fresh complete medium was added after rinsing the plates with RPMI. Then the cell culture supernatants were collected at 0, 4, 24, 48 and 72h post infection (p.i.) and the plates containing the cells were frozen. The kinetics assays were run in triplicate for both strains. For each time point, the three supernatants collected were pooled (equal volume) for further virus titration performed by hemadsorption assay on PAMs as previously described (35).

### Quantitative RT-PCR in PAMs

RNA were extracted directly from each well of infected PAMs (after harvesting the supernatant) with the NucleoSpin^®^ RNA kit (REF 740955, Macherey-Nagel, Düren, Germany) and gene expressions were determinated by reverse transcription-quantitative PCR (RT-qPCR) using the GoTaq^®^ 1-Step RT-qPCR system (Promega ref A6020). For each reaction, 80 ng of RNA in 5 µl final volume were added to the mix with experimental or control primers, according to the manufacturer’s instructions. For *IFN-α*, the sequences used were 5’GGCTCTGGTGCATGAGATGC3’ and 5’CAGCCAGGATGGAGTCCTCC3’, while 5’AGTTGCCTGGGACTCCTCAA3’ and 5’CCTCAGGGACCTCAAAGTTCAT3’ were used to quantify *IFN-β* expression, 5’ATGAAAATGGAGGTCATCTTCAAAAC3’ and 5’AAGTTTAATGACCATGAGTCTTACC3’ for P30 viral gene, 5’ACATGGCCTCCAAGGAGTAAGA3’ and 5’GATCGAGTTGGGGCTGTGACT3’ for *GAPDH*. The relative amount of IFN-α, IFN-β and P30 transcripts were normalized to the endogenous GAPDH reference and then calculated using the 2-ΔΔCt method (36).

### cDNA pre-amplification and RT-qPCR on the BioMark HD real-time PCR platform

Extracted total RNA was converted to cDNA by reverse transcription of 20 ng of RNA with iSCRIPTtm Reverse Transcription Supermix (Bio-Rad). Pre-amplification was performed with cDNA diluted 1:4 in RNAse free water using a Preamp Master Mix (Fluidigm, # 100-5581) in a T100 thermal cycler (BioRad). One µl of Preamp Master Mix was mixed with 0.5 µl of a 500 nM primer stock (**Supplementary Table 1**), 2.25 µl of H2O and 1.25 µl of the diluted cDNA. The pre-amplification thermal cycle conditions were: 95°C for 2 minutes (min) followed by 14 cycles of 95°C for 15 seconds and 60°C for 4 min. Pre-amplified cDNA was subjected to an exonuclease treatment for 30 min (Exonuclease I, New England Biolabs, #MO293) and diluted 1:5 in TE (10 mM Tris-HCI, 1.0 mM EDTA).

Real-time quantitative PCR was performed in a 48x48 Dynamic Array Integrated Fluidic Circuits (Fluidigm), combining 48 samples with 48 primer sets for 2304 simultaneous qPCR reactions. The reaction mix for each of the 48 samples was as follows: three µl of 2x SsoFast EvaGreen with Low Rox (Biorad, #172-5211), 0.3 µl of 20x DNA binding dye (Fluidigm, #100-7609) and 2.7 µl of pre-amplified cDNA. Specific primer mixes for each of the primer sets were: 3.5 µl of 2x assay loading reagent (Fluidigm), 2.8 µl of TE (10 mM Tris, 0.1 mM EDTA, pH 8.0), 0.35 µl of 100 µM forward primer and 0.35 µl of 100 µM reverse primer. 5 µl of the reaction mix and specific primer mix were dispensed into the appropriate inlets and loaded into the circuit. The thermal cycle conditions were 1 min at 95°C, followed by 30 cycles with denaturing for 5 secondes (sec) at 96°C and annealing/elongation for 20 sec at 60°C. The quantitative RT-PCR data were analyzed using the 2ΔΔCt method, where the amount of target, normalized to 3 endogenous reference genes and relative to an experimental control, is given by 2-ΔΔCt. The results are expressed as relative fold change (Fc) in comparison with the the Georgia 2007/1 at 4h p.i.

### Plasmid DNA constructs

Open reading frame (ORF)-encoding sequences from the Georgia 2007/1 strain were amplified by PCR (Thermo Scientific™ Phusion™ Plus DNA Polymerase) from infected PAMs. Amplification was performed using ORF-specific primers flanked with the Gateway cloning sites 5′-GGGGACAACTTTGTACAAAAAAGTTGGC and 5′-GGGGACAACTTTGTACAAGAAAGTTGG. PCR products were cloned by *in vitro* recombination into pDONR207 (Gateway System; Invitrogen). ORF coding sequences were subsequently transferred by *in vitro* recombination from pDONR207 into different Gateway-compatible destination vectors (see below) according to the manufacturer’s recommendations (LR cloning reaction; Invitrogen). In HEK-293T cells, GST tag and 3×FLAG tag fusions were achieved using pDEST27 (Invitrogen) and pCI-neo-3×FLAG vector, respectively (37). Expression vectors carrying genes for the constitutively active forms of RIG-I (NΔRIG-I) (38) or IRF3 (IRF3-5D) (39) and pCI-neo-3×FLAG vectors encoding MAVS or TBK1 were used to stimulate the luciferase reporter gene downstream of an IFN-β-specific promoter sequence. ORFs encoding components of our swine IFN-α/β response-dedicated library (MDA5, TRAF3, IRF3, TBK1, PKR, NEMO, STING, TRIF, IKKε, IKKα, IRF7, MAVS, RIG-I, STAT1, STAT2) and human TRAF3 were cloned in pTwist ENTR vectors by Twist Bioscience company (service delivery).

All sequences are available in the literature and Genbank references are provided in **Table 1**. ORFs from pTwist ENTR vectors were transferred into a Gateway^®^-compatible destination vector following the same protocol as previously detailed above.

**Table 1:** GenBank accession numbers for viral and cellular genes referenced in the study

Species	Gene	Genbank reference
Georgia 2007/1	MGF360-12L	CAD2068381.1
	MGF360-13L	CAD2068382.1
	MGF360-14L	CAD2068383.1
	MGF505-1R	CAD2068380.1
	MGF505-2R	CAD2068384.1
	MGF505-3R	CAD2068385.1
	MGF505-4R	CAD2068387.1
	G_ACD_00520	CAD2068386.1
Swine	IKKα	NM_001114279_1
	IKKϵ	XM_021063307.1
	IRF3	NM_213770_1
	IRF7	NM_001097428.1
	MAVS	NM_001097429_1
	MDA5	NM_001100194_1
	NEMO	NM_001113053.1
	PKR	XM_021085862.1
	RIG-I	NM_213804_2
	STAT1	NM_213769_1
	STAT2	NM_213889.1
	STING	NM_001142838.1
	TBK1	NM_001105292_1
	TRAF3	XM_005666444.3
	TRIF	NM_001315738.2
Human	MAVS	NM_020746.5
	TBK1	NM_013254.4
	TRAF3	NM_003300.4

### Luciferase reporter gene assays

HEK-293T were plated in 24-well plates (5 × 10^5^ cells per well). One day later, HEK-293T cells were co-transfected (JetPRIME; Polyplus) with IFN-β-pGL3 or pISRE-Luc plasmid (0.3 μg/well; Stratagene) that contain the firefly luciferase reporter gene downstream of an IFN-β-specific promoter sequence or the ISRE enhancer element upstream, respectively. Cells were simultaneously co-transfected with the pRL-CMV reference plasmid (0.03 μg/well; Promega) and the empty pCI-neo-3×FLAG expression vector (0.3 μg/well) or encoding proteins as specified. When specified, cells were transfected with 0.1 μg/well of poly(dA:dT) (Invivogen) or treated with 1 x 10^3^ IU/ml of recombinant IFN-β (PBL Assay Science) 24h after transfection. After 24h post-IFN-β-stimulation or 48h post-transfection, the cells were lysed (Passive lysis buffer, Promega), and both firefly and *Renilla* luciferase activities in the lysate were determined using the Bright-Glo and Renilla-Glo luciferase assay systems (Promega), respectively. The reporter activity was calculated as the ratio of firefly luciferase activity to the reference *Renilla* luciferase activity. All graphs show mean values and include error bars indicating the standard deviations (SD).

### Nanoluciferase-2-hybrid (N2H) complementation assays

The N2H protocol complementation assay was adapted from the report of Choi et al. (40). The NanoLuciferase fragments 1 (aa1-65aa) and 2 (aa66-aa171) were linked to the N-terminal (pDEST-N2H-N1, -N2) extremity of the tested protein. HEK-293T were plated in white 96-well plates (1 × 10^5^ cells per well, Greiner Bio-One). HEK-293T cells were co-transfected (JetPRIME; Polyplus) 24h later with pDEST-N2H-N1 encoding MGF505-4R and pDEST-N2H-N2 alone or fused to the indicated swine cellular protein (0.1 μg of each plasmid/well). After 24h, cells were lysed (Passive lysis buffer, Promega) and then incubated with a solution of hikarazine-103 (Z103) provided by Dr Yves L. Janin (41, 42). Enzymatic activity of the NanoLuciferase, quantified as bioluminescence, was measured using a GloMax^®^ luminometer (Promega).

### GST pulldown experiments and Western blot analysis

HEK-293T cells were plated in 6-well plates (2 × 10^6^ cells) before being transfected, 24h later, (JetPRIME; Polyplus) with pDEST 27 (Invitrogen) expressing either GST alone (0.5 μg) or GST-MGF505-4R (0.5 μg) and with 0.5 μg of pCI-neo-3xFLAG vector encoding human or swine TRAF3. After 48h, cells were collected in PBS and then lysed on ice in lysis buffer (20 mM morpholinepropanesulfonic acid [MOPS]-KOH [pH 7.4], 120 mM KCl, 0.5% Igepal, 2 mM β-mercaptoethanol, supplemented with complete protease inhibitor cocktail [Roche]) for 20 min. Cell lysates were clarified by centrifugation at 14,000 × g for 30 min. For pulldown analysis, protein extracts were incubated for 2h at 4°C on a spinning wheel with 30 μL of glutathione-Sepharose beads (Amersham Biosciences). Beads were then washed 3 times for 5 min with ice-cold lysis buffer and on a spinning wheel, and proteins were recovered by boiling in denaturing loading buffer (Invitrogen).

Purified complexes and protein extracts were boiled at 95°C for 5 min and resolved by SDS-polyacrylamide gel electrophoresis (SDS-PAGE) on 4 to 12% NuPAGE bis-Tris gels with Bolt morpholineethanesulfonic acid (MES) SDS running buffer and transferred to a nitrocellulose membrane under wet conditions (Invitrogen). GST- and 3×FLAG-tagged proteins were detected with a rabbit polyclonal anti-GST antibody (1:2,500; Sigma-Aldrich) and a mouse monoclonal horseradish peroxidase (HRP)-conjugated anti-FLAG antibody (M2; 1:10,000; Sigma-Aldrich), respectively. Secondary anti-rabbit HRP-conjugated antibody was purchased from Invitrogen (1:5,000). Nitrocellulose membrane was then incubated with a peroxidase substrate (Clarity Western ECL substrate; Bio-Rad) and visualized with the ChemiDoc MP imaging system (Bio-Rad).

### Confocal fluorescence microscopy assays

Plates (24-well; Ibidi, BioValley) were seeded with HeLa cells (9 × 10^4^ cells per well). One day later, cells were transfected with 200 ng of pCherry-C1 encoding human TRAF3 and/or 800 ng of pEGFP-C1 expressing MG505-4R. After 24h, cells were fixed with a 4% paraformaldehyde (PFA) solution (Electron Microscopy Sciences) for 30 minutes, treated with PBS-glycine (0.1 M) for 5 minutes, and incubated with a solution containing Hoechst 33342 dye (1/10,000) (Life technologies). Preparations were visualized using a Leica DMI 8 confocal microscope (×40 magnification).

### Statistical analyses

p-values are a result of two-tailed t-tests and R square (R) values are a result of linear regression tests. Both p-values and R square (R) values were calculated using Prism 7, version 7.0a. All graphs represent the mean, and include error bars of the standard deviation.

## Results

### Differential *in vivo* production of IFN-α by Georgia 2007/1 and ASFV-989

**Figure 1:**
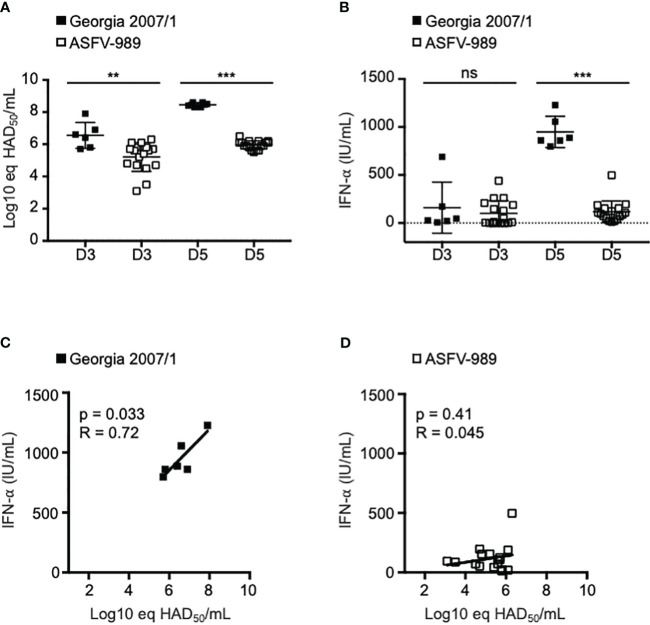
Assessment of IFN-α levels in pigs infected with Georgia 2007/1 and ASFV-989. SPF pigs were i.m. infected with 1 x 10^3^ HAD_50_ of the Georgia 2007/1 (black squares, n = 6) and ASFV-989 strains (white squares, n = 17). **(A)** At the indicated days post-infection (D3 and D5), blood samples were collected and ASFV viremia (Log10 eq HAD50/mL) was measured using a qPCR test (33) that detect both Georgia 2007/1 and ASFV-989 strains. **(B)** The protein levels of IFN-α were quantified in the sera by ELISA and expressed as units/ml (IU/ml). Correlations were determined by comparing P72 levels at D3 and the IFN-α levels at day D5 (C, Georgia 2007/1 and D, ASFV-989. **, p < 0.005, ***, p < 0.0005 and “ns” for non significant.

To evaluate the IFN-α/β response following infection by Georgia 2007/1 and ASFV-989, SPF pigs were i.m. infected with 1 x 10^3^ HAD_50_ of each strain. This dose was lethal in all pigs infected with Georgia 2007/1 by day 5-6 p.i., whereas pigs infected with ASFV-989 strain displayed limited symptoms (32). As a measure of viral load, we determined the expression of the P72 viral gene by qPCR in the blood samples collected from pigs infected with Georgia 2007/1 and ASFV-989 strains (33). In these conditions, P72 expression in pigs infected with Georgia 2007/1 was significantly increased by more than 1.0 and 2.0 log 10 relative to those infected with ASFV-989 at days 3 (D3) and 5 p.i. (D5), respectively (**Figure 1A**). This suggests that the parental Georgia 2007/1 strain replicates more efficiently than its attenuated counterpart *in vivo*. In the sera from the same samples, we determined the protein level of IFN-α that is known to be highly expressed during ASFV infection (20). We did not see any significant increase in IFN-α at day 3. However, at day 5, the protein levels of IFN-α were significantly increased in pigs infected with Georgia 2007/1 compared to those infected with ASFV-989 (**Figure 1B**). Indeed, there is a statistically significant positive correlation between the P72 expression of Georgia 2007/1 at day 3 and the high level of IFN-α at day 5 (**Figure 1C**). In contrast, ASFV-989 infected pigs did not show any correlation between viral replication and IFN-α levels (**Figure 1D**). Taken together, these observations suggest that high ASFV replication results in a significant IFN response in pigs infected with the virulent Georgia 2007/1 strain, but not for its ASFV-989 attenuated counterpart.

### Comparison of viral replication dynamics between Georgia 2007/1 and ASFV-989 in PAMs

**Figure 2:**
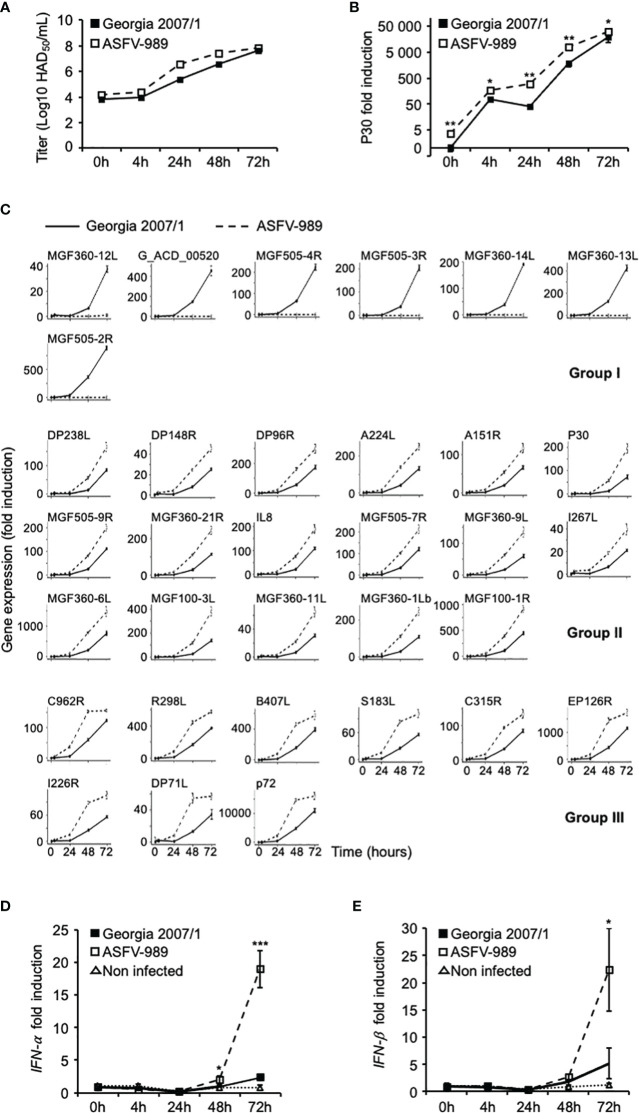
The attenuated ASFV-989 strain showed increased replication and elevated IFN-α and IFN-β in porcine alveolar macrophages. PAMs were infected with Georgia 2007/1 or ASFV-989 at a MOI of 2. Black and white squares correspond to Georgia 2007/1 and ASFV-989, respectively, while white triangles correspond to non-infected PAMs **(D, E)**. **(A)** At the indicated times, the supernatants were collected and used to evaluate the virus titers. **(B)** The expression level of the P30 viral gene was measured by RT-qPCR and normalized to that of *GAPDH*. Data are presented as a fold increase relative to PAMs infected with Georgia 2007/1 at 0h p.i. **(C)** Expressions levels of 33 viral genes were measured and shown as a fold increase relative to the Georgia 2007/1 at 4h p.i. The expression levels of *IFN-α* **(D)** and *IFN-β* **(E)** were evaluated by RT-qPCR. *, p < 0.05, **, p < 0.005 and ***, p < 0.0005. Data are representative of three independent experiments.

To better characterize the differences between the Georgia 2007/1 and ASFV-989 strains, we followed the kinetics of viral replication in infected PAMs over three days using a MOI of 2. The virus was harvested from the pool of supernatants and titrated at each kinetics time. The results showed that similar virus titers (≅ 1 x 10^4^ HAD_50_/ml) were obtained at 0h p.i., confirming that PAMs were infected with the same amount of virus for both viral strains (**Figure 2A**). From 4 to 48h p.i., higher titers were obtained from cells infected with ASFV-989, suggesting a faster viral replication for the attenuated strain compared to its parental strain. However, both viral strains exhibited similar viral titers at 72h p.i. (between 6 x 10^7^ and 8 x 10^7^ HAD_50_/ml). To confirm this result, total RNAs were extracted from infected PAMs and the expression of the P30 viral gene was determined by RT-qPCR. We observed higher P30 mRNA levels in cells infected with ASFV-989 in early time points, but similar levels at 72h, consistent with the results of infectious viral titers (**Figure 2B**). As a complementary approach, we used a medium-throuput qPCR approach based on microfluidics technology to measure simultaneously the transcripts levels of 33 ASFV genes at 0, 4, 24, 48 and 72h p.i. Viral gene selection was guided by their absence in ASFV-989 and their known or hypothesized roles in ASFV virulence. In contrast to Georgia 2007/1, no transcripts were detected in ASFV-989 infected cells for MGF360-12L, -13L, -14L, MGF505-2R, -3R, -4R and G_ACD_00520 (**Figure 2C**, group I), which is consistent with its known genetic deletion (32). Most interestingly, the expression kinetics showed two other groups of viral genes. The expression kinetics of group II genes were characterized by a sustained increase in transcripts level, while those of group III seemed to reach a plateau starting from 48h p.i. and were exclusively observed in the case of ASFV-989. In both cases, the expression level of genes from ASFV-989 were higher than those from Georgia 2007/1 at all time points confirming the difference in virus replication between these two ASFV strains in PAMs.

The same RNA samples were used to measure the expression levels of *IFN-α* and *IFN-β*. As shown in **Figures 2D, E**, expression levels of *IFN-α* and *IFN-β* mRNAs in PAMs infected with ASFV-989 at 72h p.i. were increased by more than 8 and 4-fold, respectively, relative to those infected by Georgia 2007/1. Although we cannot exclude the possibility that the increased expression levels of *IFN-α* and *-β* could be caused by the higher viral replication of ASFV-989 in PAMs, we hypothesized that gene deletions in the ASFV-989 strain might also explain its inability to efficiently control the IFN-α/β response *in vitro*.

### MGF505-4R inhibits the induction of the IFN-α/β signaling pathway

**Figure 3:**
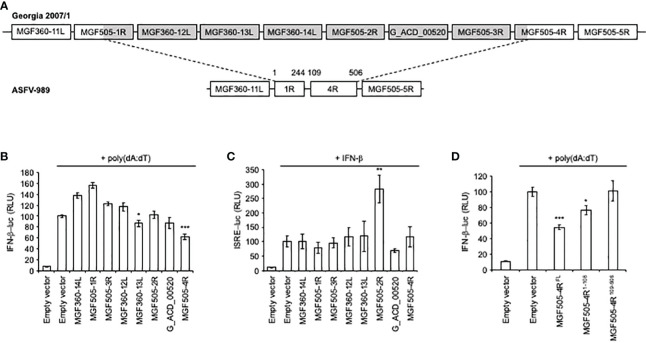
Activation of the IFN-β and ISRE promoters in cells expressing ASFV proteins. **(A)** Schematic representation of the ASFV genes at the site of the deletion. The numbers indicate amino acids (aa) positions. **(B)** HEK-293T cells were co-transfected with IFN-β-pGL3 and pRL-CMV reference plasmids, poly(dA:dT) and pCI-neo-3×FLAG expression vectors encoding 3×FLAG alone or fused to the indicated ASFV ORFs. After 48h, the relative luciferase activity was determined. **(C)** Same experiment as **(B)** except that transfected cells were stimulated after 24h with 1000 IU/ml of IFN-β and expression of the luciferase reporter construct controlled by ISRE repeats (pISRE-Luc) was quantified 24h later. **(D)** Same experiment as **(B)** but cells were co-transfected with full-length (FL) or the indicated fragments of MGF505-4R. All experiments were achieved in triplicate. Data represent means ± SD and are representative of three independent experiments. *, p < 0.05, **, p < 0.005 and ***, p < 0.0005.

Our previous genomic study has identified eight genes of the MGF360 and 505 as being fully (MGF360-12L, -13L, -14L, MGF505-2R, -3R and G_ACD_00520) or partially (MGF505-1R and -4R) deleted in the ASFV-989 attenuated strain compared to its parental Georgia 2007/1 (**Figure 3A**). As a first approach to understand the possible correlation between attenuation of ASFV-989 and its inability to control the IFN-α/β response in infected PAMs, we individually tested the ability of these eight ASFV proteins to modulate the IFN-α/β pathway using luciferase gene reporter assays. HEK-293T cells were transfected with an IFN-β-luciferase reporter gene in the presence of MGF360-12L, -13L, -14L, MGF505-1R, -2R, -3R, -4R or G_ACD_00520 and stimulated with poly(dA:dT), which mimics viral double-stranded DNA. As shown in **Figure 3B**, significant inhibition of IFN-β promoter activity was observed only in cells expressing the viral proteins MGF360-13L and MGF505-4R. However, none of the ASFV proteins has shown any antagonism effect on ISRE-luciferase gene expression (**Figure 3C**) in IFN-β-stimulated cells. Although we have no explanation for this phenomenon, it should be noted that MGF505-2R positively impacted (2.5-fold increase, relative to the positive control, empty vector + IFN-β). MGF360-13L has been recently described to inhibit the IFN-α/β response (43). In contrast, the inhibition of the IFN-α/β induction pathway by MGF505-4R had not yet been identified and suggest a new function for this viral protein that we have decided to investigate.

As MGF505-4R was partially deleted in the ASFV-989 strain, we tested separately the conserved (MGF505-4R^109-506^) and deleted region (MGF505-4R^1-108^) in ASFV-989 using our IFN-β-luciferase reporter assay (**Figure 3D**). Inhibition of IFN-α/β signaling was observed with MGF505-4R^1-108^, but was only partial when compared with full-length MGF505-4R. Surprisingly, MGF505-4R^109-506^ was unable to block the IFN-β promoter activity. This may account for the high productions of IFN-α and IFN-β in PAMs infected with the ASFV-989 strain, and for the attenuation phenotype of that strain.

### MGF505-4R inhibits IFN-α/β signaling downstream of RIG-I and MAVS

**Figure 4:**
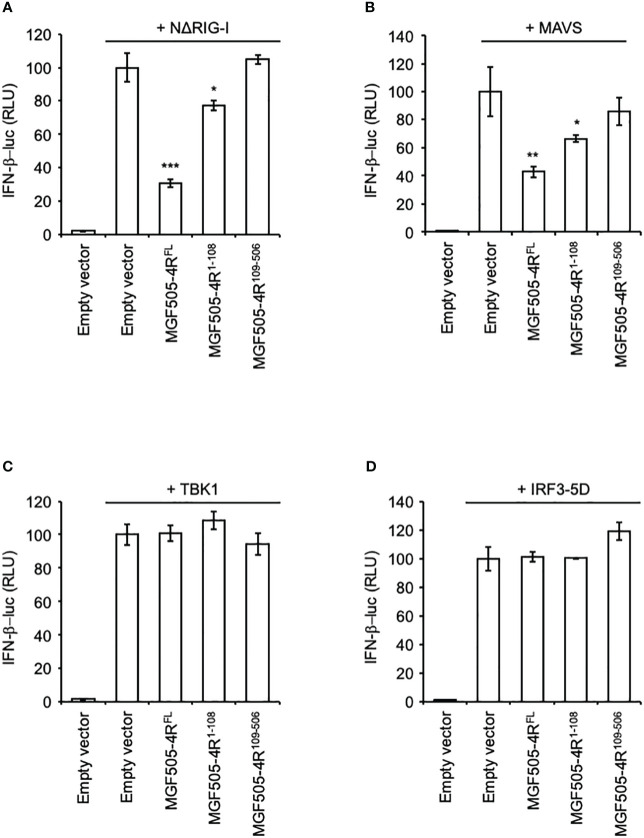
Effects of MGF505-4R on IFN-β promoter upon activation with different components of the IFN-α/β induction pathway. HEK-293T cells were co-transfected with IFN-β-pGL3 and pRL-CMV reference plasmids and pCI-neo-3×FLAG expression vectors encoding 3×FLAG alone or fused to MGF505-4R^FL^, MGF505-4R^1-108^ or MGF505-4R^109-506^. The IFN-β promoter was activated by overexpressing NΔRIG-I **(A)**, MAVS **(B)**, TBK1 **(C)** or IRF3-5D **(D)**. All experiments were achieved in triplicate. Data represent means ± SD and are representative of three independent experiments. *, p < 0.05, **, p < 0.005 and ***, p < 0.0005.

To further decipher at which stage of the IFN-α/β signaling pathway MGF505-4R acts, we overexpressed NΔRIG-I (constitutively active N-terminal CARDs of RIG-I), MAVS, TBK1, or IRF3-5D (a constitutively active form of IRF3) to activate the IFN-β promoter. As shown in **Figures 4A, B**, MGF505-4R was able to efficiently reduce the stimulating effect of NΔRIG-I or MAVS. However, this inhibitory effect was lost after overexpression of TBK1, or IRF3-5D (**Figures 4C, D**) suggesting that MGF505-4R interferes at least with the signal transduction upstream of the TBK1/IKKϵ complex after activation of RIG-I and MAVS. Similar to what is seen with poly(dA:dT) stimulation, the MGF505-4R^1-108^ region conserved its partial inhibitory effects on IFN-α/β signaling downstream of RIG-I and MAVS whereas MGF505-4R^109-506^ was unable to do so (**Figures 4A, B**).

### MGF505-4R interacts with TRAF3

**Figure 5:**
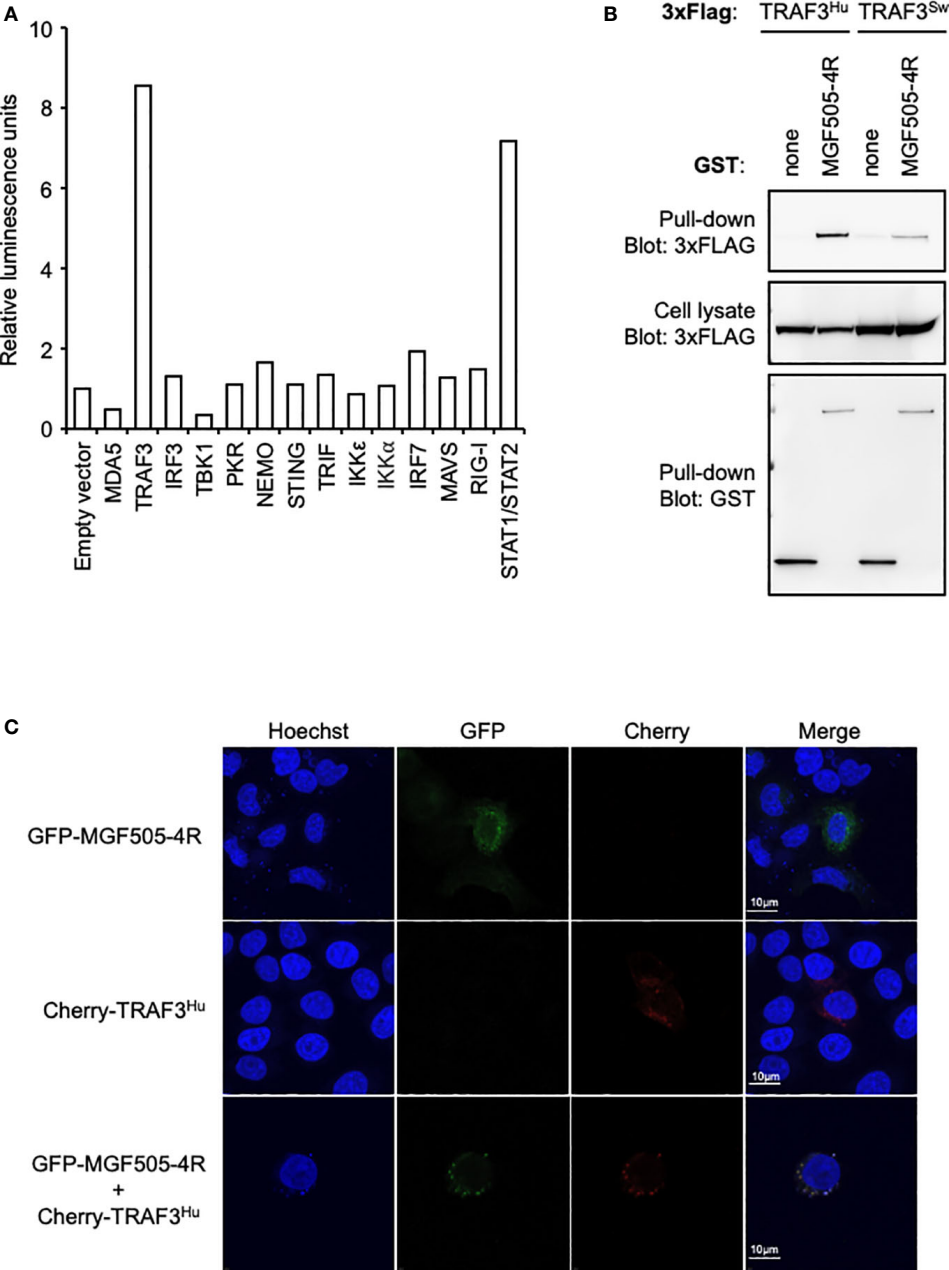
MGF505-4R interaction with TRAF3. **(A)** HEK-293T cells were co-transfected with pDESTN2H-N1 encoding the fragment N1 of the NanoLuciferase (aa1-aa65) fused to MGF505-4R and pDESTN2H-N2 encoding the fragment N2 of the NanoLuciferase (aa66-aa171) alone or fused to the indicated swine cellular protein. STAT1/STAT2 interaction was used as positive control. In this condition, STAT1 and STAT2 were expressed in pDESTN2H-N1 and pDESTN2H-N2, respectively. After 48h, cells were lysed, the bioluminescence was measured and data are presented as a fold increase relative to the condition where pDESTN2H-N1-MGF505-4R and pDESTN2H-N2 empty vector were co-transfected. **(B)** HEK-293T cells were transfected with expression vectors encoding GST alone or fused to MG505-4R and tested for the interaction with either human or swine TRAF3. Total cell lysates were prepared 48h post-transfection (cell lysate; middle panel), and co-purifications of indicated cellular proteins were assayed by pull-down using glutathione-sepharose beads (pull-down; upper panel). GST-tagged MG505-4R was detected by immunoblotting using anti-GST antibody (pull-down; lower panel), while TRAF3 was detected with an anti-3xFLAG antibody. **(C)** HeLa cells were co-transfected with pCherry-C1 and pEGFP-C1 plasmids encoding human TRAF3 and MG505-4R, respectively. 24 h after, cells were fixed and labeled with the dye Hoechst 33258 to stain nuclei. Intracellular localization of Hoechst-stained nuclei (blue), TRAF3 (red) and MG505-4R (green) were visualized by confocal fluorescence microscopy (×40 magnification). Scale bars represent 10 µM.

**Figure 6:**
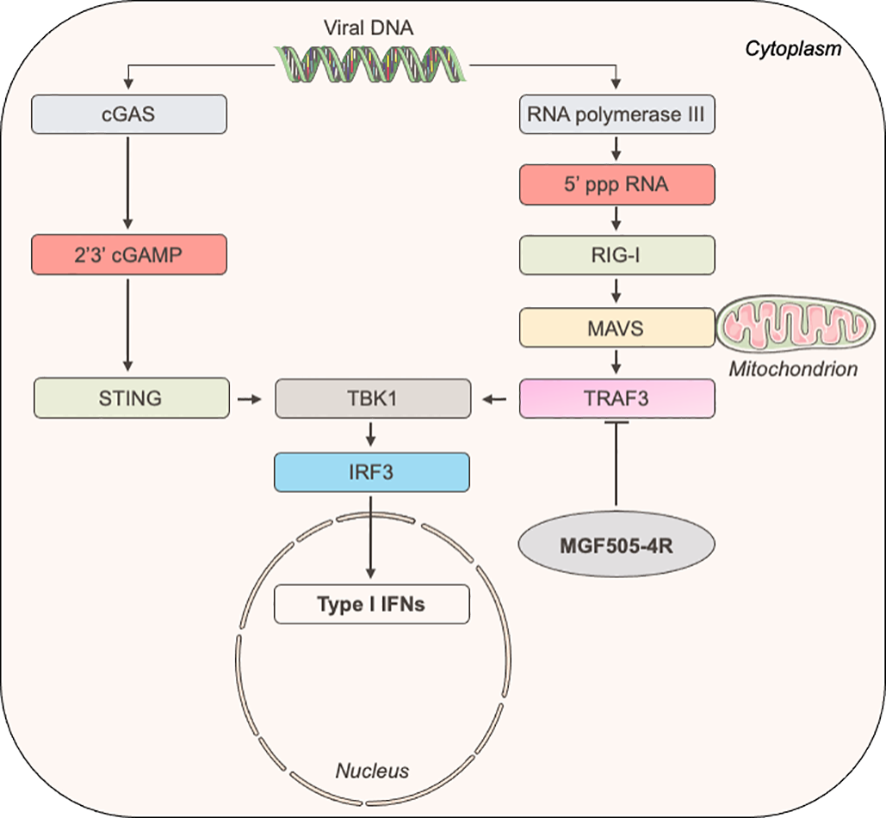
Schematic representation of the induction of IFN-α/β signaling upon the recognition of ds viral DNA.

To better understand how MGF505-4R inhibits the IFN-α/β induction pathway at the molecular level, we designed a NanoLuciferase two-hybrid (N2H) assay to screen a swine IFN-α/β response-dedicated library, which contains a set of proteins involved in the induction of IFN-α/β signaling: MDA5, TRAF3, IRF3, TBK1, PKR, NEMO, STING, TRIF, IKKε, IKKα, IRF7, MAVS and RIG-I. Our N2H system is based on the reconstitution of the NanoLuciferase where MGF505-4R and cellular proteins were expressed in fusion with the N1 or N2 fragment of the NanoLuciferase, respectively. The heterodimer STAT1/STAT2 was used as positive control while the negative control corresponded to the condition where the N1-MGF505-4R construct was co-transfected with the N2 empty vector. As shown in **Figure 5A**, only the interaction between MGF505-4R and TRAF3 exhibited a luminescence score equivalent (8.5) to the positive control (7.2). To validate this interaction, 3×FLAG-tagged TRAF3 was co-expressed in HEK-293T with GST-tagged MGF505-4R and purified with glutathion-sepharose beads. As shown in **Figure 5B**, MGF505-4R co-purified with both human and swine TRAF3. To evaluate how the interaction between MGF505-4R and TRAF3 might influence their respective subcellular localizations, we conducted fluorescence microscopy experiments using HeLa cells. TRAF3 was co-expressed in fusion downstream of the red fluorescent protein Cherry with MGF505-4R tagged with GFP. When expressed independently, both TRAF3 and MGF505-4R exhibited a punctiform distribution within the cytoplasm. Upon co-transfection, Cherry-tagged TRAF3 colocalized with GFP-tagged MGF505-4R in the cytoplasm, displaying similar distribution patterns (**Figure 5C**). TRAF3 plays a central role in regulating the IFN-α/β signaling, notably by acting as a platform for mitochondrial antiviral signaling (MAVS), NF-κB essential modulator (NEMO) and TANK-binding kinase/the IκB kinaseϵ (TBK1/IKKϵ) kinase complex. Therefore, binding to TRAF3 represents a potential molecular mechanism underlying the inhibition of the IFN-α/β pathway by MGF505-4R (**Figure 6**).

## Discussion

ASFV clinical manifestations are highly variable, ranging from asymptomatic infections to lethal hemorrhagic fevers. This variability is due to several factors related both to the infected host and the viral strains. Highly virulent ASFV strains lead to severe disease, often accompanied by mortality rates nearing 100%. On the other hand, attenuated ASFV isolates induce only mild disease with few, mostly unspecific, symptoms. These diverse clinical outcomes may arise from variations in the immune responses to ASFV, themselves relying on the activation of the IFN-α/β signaling pathway (26). In contrast to the Georgia 2007/1 virulent strain, we have demonstrated *in vitro* that macrophages infected with the ASFV-989 attenuated strain induced elevated levels of IFN-α and IFN-β transcripts at 72h p.i. While increases in *IFN-α/β* gene expression occur at a late stage, our findings are reminiscent to what has been observed by others, showing the same significant difference in IFN-α/β production between avirulent/low virulent and high virulent ASFV strains (15, 21, 28, 44, 45). This delayed induction of *IFN-α* and *IFN-β* gene expression could be attributed to the variation in the numbers and identities of MGF360 and 505 genes that are absent in different attenuated ASFV strains. Moreover, the same gene deletions might contribute to potential variations in the kinetics of viral replication and the IFN-α/β response, depending on the strains and/or genotypes. Intriguingly, pigs infected with Georgia 2007/1 showed higher IFN-α levels than those infected by ASFV-989, in contrast to our *in vitro* observations. One possible explanation could be that the high levels of IFN-α at day 5 clearly coincided with its important viral replication at day 3 (**Figure 1C**), as previously suggested by Golding et al. (20). This may also reflect the potential differences between the types of macrophages that ASFV infects *in vivo* and the PAM *in vitro* model. In response to ASFV infection *in vivo*, IFN-α may also be alternatively secreted by other mononuclear phagocytes of the myeloid lineage, such as monocytes and dendritic cells (46–48).

The differential impact of these two ASFV strains on IFN-α/β production led us to subsequently investigate whether proteins encoded by the eight genes deleted in ASFV-989 play a role in modulating the IFN-α/β signaling pathway. While the expression of some viral genes, such as MGF360-12L, -14L, MGF505-1R, -2R, -3R and G_ACD_00520 seems to have no effect on the induction of IFN-α/β signaling downstream of poly(dA:dT) stimulation, MGF360-13L and MGF505-4R significantly inhibit this pathway. MGF360-13L has recently been described as an inhibitor of the cGAS-STING-mediated IFN-α/β pathway (43). Nevertheless, the absence of cGAS and STING in HEK-293T (49) implies that the inhibition we observed for MGF360-13L is independent of the cGAS-STING axis. Intriguingly, other studies have shown that MGF360-14L and MGF360-12L can also block IFN-α/β production by promoting IRF3 degradation (50) and modulating the NF-κB signaling pathway (51, 52), respectively. Moreover, the deletion of MGF360-12L from Georgia 2007/1 virus from which the K145R viral gene was also deleted led to a higher level of IFN-a/b production *in vitro* compared to the parental strain (53). However, these findings do not necessarily contradict our current data as the experimental conditions were not identical. Unexpectedly, MGF505-4R was found to inhibit the IFN-α/β induction pathway, revealing a novel function for this viral protein in immune evasion.

Next, we identified by NanoLuciferase two-hybrid assays and validated by co-affinity purification experiments the interaction between MGF505-4R and TRAF3. The latter plays a crucial role in the regulation of the IFN-α/β induction pathway mediated by Toll-like (TLR) receptors and RIG-I-like receptors (RLR) families (54, 55). Upon activation of RIG-I, TRAF3 is recruited by MAVS, then promotes the ubiquitination of K63, providing a platform for NF-kappa-B essential modulator (NEMO) and TBK1/IKKϵ complex to activate IRF3 and initiates IFN-α/β expression (56–59). As demonstrated in a recent study by Ran et al. (31), the AT-rich DNA genome of ASFV can trigger IFN-α/β signaling through the RNA Polymerase III-RIG-I pathway. Moreover, ASFV-I267L was reported in this article to disrupt this pathway by preventing the activation of RIG-I. The interaction of MGF505-4R with TRAF3 is thus consistent with the fact that MGF505-4R is able to inhibit the IFN-α/β induction downstream of RIG-I and MAVS but not TBK1 and IRF3. Therefore, this interaction represents another mechanism developed by ASFV to interfere with the RNA Polymerase III-RIG-I axis. As TRAF3 is essential for virus-induced activation of the IFN-α/β system, some viruses have evolved to gain multiples strategies in order to control the IFN-α/β pathway by directly targeting TRAF3. The M proteins of Severe acute respiratory syndrome coronavirus 1 (SARS-CoV-1) and Middle East respiratory syndrome coronavirus (MERS-CoV) as well as the X protein from hepatitis B virus and VP3 from *Avibirnavirus* have been reported to bind TRAF3, disrupting the TRAF3–TBK1 association and subsequently suppressing IFN-α/β production (60–64). Influenza A NS1 protein was found to interact with TRAF3 preventing its activation and association with MAVS (65, 66). Other viruses such as Epstein-Barr virus or human papillomavirus have also been shown to target TRAF3 but the possible contribution to the IFN-α/β immune evasion remains to be established (67, 68). In the future, it will be essential to elucidate the molecular mechanisms through which the interaction of MGF505-4R with TRAF3 could alter the MAVS-TRAF3-TBK1 complex.

The cGAS-STING-mediated IFN-α/β signaling is currently the most studied pathway for understanding the detection and immune evasion of ASFV. However, some studies have indicated a potential role of TRAF3 in the cGAS-STING-mediated IFN-α/β signaling pathway, as evidenced by its interaction with STING (69–71). A noteworthy study examining the ability of human enterovirus A71 to control this pathway proposed the significance of TRAF3 in facilitating the STING-TBK1 interaction and subsequent activation of TBK1 via phosphorylation (70). Consequently, further investigations are warranted to determine whether MGF505-4R can potentially counteract cGAS-STING-mediated IFN-α/β signaling through its interaction with TRAF3.

Interestingly, our findings indicate that MGF505-4R^1-108^ retained the ability to only partially inhibit IFN-α/β induction in comparison to the full-length MGF505-4R protein. However, the conserved fragment (MGF505-4R^109-506^) in the ASFV-989 attenuated strain was observed to compromise the inhibitory function of MGF505-4R. In addition to other deletions within the MGF360 and 505 regions, MGF505-4R has also been partially deleted in the genome of the ASFV isolate Benin 97/1, from which higher levels of IFN-β mRNA were observed compared to its parental virulent strain (21). The identification of MGF505-4R as a novel IFN antagonist, coupled with the loss-of-function phenotype of MGF505-4R^109-506^, provide molecular basis underlying the attenuation of ASFV-989.

While an increasing number of reports have illustrated how ASFV has evolved various mechanisms to counteract IFN-α/β signaling (16, 17, 25, 27, 72–74), this marks the first description of ASFV targeting TRAF3. Altogether, this emphasizes the evolution of ASFV to encode numerous viral proteins with redundant functions in immune evasion. Collectively, deciphering molecular virus-host protein interactions and mechanisms underlying the control of the innate immune response by ASFV will constitute an important theoretical basis to reveal new factors of virulence and develop future rationally designed ASFV vaccines (75).

## Data availability statement

The original contributions presented in the study are included in the article/**Supplementary Material**. Further inquiries can be directed to the corresponding author.

## Ethics statement

Ethical approval was not required for the studies on humans in accordance with the local legislation and institutional requirements because only commercially available established cell lines were used. The animal study was approved by the ethics committee N°16 (authorization number 19-018#19585) and authorized by the French Ministry of Research (project n°2019030418445731). The study was conducted in accordance with the local legislation and institutional requirements.

## Author contributions

JD: Conceptualization, Investigation, Methodology, Validation, Visualization, Writing – original draft, Writing – review & editing. MD: Conceptualization, Investigation, Methodology, Validation, Visualization, Writing – original draft, Writing – review & editing. EH: Investigation, Methodology, Validation, Visualization, Writing – review & editing. PD: Investigation, Methodology, Validation, Visualization, Writing – review & editing. AF: Investigation, Methodology, Validation, Visualization, Writing – review & editing. AL: Investigation, Methodology, Validation, Visualization, Writing – review & editing. IF: Investigation, Methodology, Validation, Visualization, Writing – review & editing. GK: Writing – review & editing, Methodology, Conceptualization, Investigation, Visualization. FR: Conceptualization, Investigation, Methodology, Supervision, Validation, Visualization, Writing – review & editing. IC: Conceptualization, Investigation, Methodology, Supervision, Validation, Visualization, Writing – original draft, Writing – review & editing. OB: Visualization, Writing – review & editing, Conceptualization, Investigation, Methodology, Supervision, Validation. DV: Conceptualization, Investigation, Methodology, Supervision, Validation, Visualization, Writing – review & editing. M-FP: Conceptualization, Funding acquisition, Investigation, Methodology, Project administration, Resources, Supervision, Validation, Visualization, Writing – original draft, Writing – review & editing. GC: Conceptualization, Funding acquisition, Investigation, Methodology, Project administration, Resources, Supervision, Validation, Visualization, Writing – original draft, Writing – review & editing.
